# On the possibility of using polycrystalline material in the development of structure-based generic assays

**DOI:** 10.1107/S090744490900256X

**Published:** 2009-03-19

**Authors:** Marc Allaire, Natalia Moiseeva, Cristian E. Botez, Matthew A. Engel, Peter W. Stephens

**Affiliations:** a National Synchrotron Light Source, Brookhaven National Laboratory, Upton, NY 11973-5000, USA; b Department of Physics and Astronomy, Stony Brook University, Stony Brook, NY 11794-3800, USA; c Department of Biomedical Engineering, Stony Brook University, Stony Brook, NY 11794-2580, USA

**Keywords:** protein–ligand complexes, assays, powder diffraction, lysozyme

## Abstract

The discovery of ligands that bind specifically to a targeted protein benefits from the development of generic assays for high-throughput screening of a library of chemicals. Protein powder diffraction (PPD) has been proposed as a potential method for use as a structure-based assay for high-throughput screening applications. Building on this effort, powder samples of bound/unbound states of soluble hen-egg white lysozyme precipitated with sodium chloride were compared. The correlation coefficients calculated between the raw diffraction profiles were consistent with the known binding properties of the ligands and suggested that the PPD approach can be used even prior to a full description using stereochemically restrained Rietveld refinement.

## Abstract 2

The correlation coefficients calculated between raw powder diffraction profiles can be used to identify ligand-bound/unbound states of lysozyme.

## 1. Introduction

The availability of gene sequences, such as the completion of the Human Genome Project, has revolutionized our understanding of life sciences at a molecular level. From sequences, we can generate a list of all the potential components of the cell. This list includes the entire possible open reading frame that could generate proteins. A grand challenge in the genomic era is to elucidate the function of these hypothetical proteins. In addition, for some of these proteins we can anticipate multiple functions, which could also involve more than one biological component. It is imperative to pursue the development of methods to characterize the role of proteins.

Numerous approaches have been developed to identify the functions of hypothetical proteins. Major developments in bio-informatics have provided tools for the analysis and comparison of gene sequences. The concept behind this approach is to infer the potential function of hypothetical proteins from their sequence homology with proteins of known function. This method is extremely powerful, especially for recognizing functions when comparing sequences from different species. Another approach is structural genomics, which aims at identifying the three-dimensional structures of most proteins that are easily obtainable from knowledge of their corresponding DNA sequences. The long-range goals of this initiative are to find all possible protein folds and to provide structural descriptions of (hypothetical) proteins. In this approach, the functions of a protein can be inferred from structural homology with another protein structure of known functionality. Nonetheless, there are a number of ‘orphan’ structures for which no known function can be assigned. In all cases the functions of these proteins remain to be further characterized *in vitro* and, more importantly, *in vivo*.

In recent years, chemical genetics has been proposed for the study of gene-product functions in the cellular or organismal context using exogenous ligands. In this approach, small molecules that bind specifically to targeted proteins are used to alter protein functions, enabling a kinetic analysis of potential phenotypes *in vivo*. In  order to be successful, the chemical genetics approach requires the identification of protein ligands. The strategy employed for the discovery of ligands is similar to that exploited by the pharmaceutical industry in the development of new drugs. It consists of high-throughput screening (HTS) of a library of chemicals (>100 000 compounds) using a particular assay that can identify the specificity of a compound for binding to a target protein. Optimizing the chemistry of an initial hit is generally required in order to increase the affinity of a ligand for its binding partner. A major limitation of the chemical genetics approach is that the function of the target protein is not known *a priori* and therefore an HTS generic assay should be used.

Generic assays take advantage of the biophysical properties of proteins when binding to a ligand. A well known example is the release of thermodynamic binding energy during the formation of protein–ligand complexes. Alternative approaches could use structure-based assays, such as a change in diffraction in macromolecular crystallography. This method of screening chemicals is limited by the tedious preparation of the large number of crystals required for HTS applications. Recently, protein powder diffraction (reviewed in Von Dreele, 2003 ▶; Margiolaki & Wright, 2008 ▶) has been proposed as a structure-based method that could facilitate this step. Pioneering work by R. B. Von Dreele has identified the potential for extracting structural information from protein powder diffraction profiles (Von Dreele *et al.*, 2000 ▶) and for the identification of ligand-bound states of lysozyme (Von Dreele, 2001 ▶, 2005 ▶; D’Amico & Von Dreele, 2006 ▶). Building on this effort, we report here that differences between bound and unbound states of lysozyme can be recognized by comparison of the powder diffraction profiles even prior to a full description following stereochemically restrained Rietveld refinements (Rietveld, 1969 ▶; Von Dreele, 1999 ▶; Larson & Von Dreele, 2004 ▶).

## 2. Experimental

The promise of protein powder diffraction is that it has the potential to distinguish bound from unbound forms of a protein when mixed with different ligands. The rationale for this experiment is based on the idea that the diffraction of protein powder would differ when directly comparing the powder profiles of unbound and ligand-bound states of lysozyme. Lysozyme hydrolyzes the β(1–4) glycosidic bond between *N*-acetylmuramic acid (NAM) and *N*-acetylglucosamine (NAG) residues in certain polysaccharides. In this experiment, we take advantage of the known properties of lysozyme to bind NAG and NAG_3_ and use glucose as a negative control (Rupley *et al.*, 1967 ▶).

In an initial step, we prepared homogenous crystalline powder from soluble lysozyme. An obvious approach in producing polycrystalline powder is to increase the number of nucleation sites by having a super-saturated solution compared with single-crystal growth conditions. Hen egg-white lysozyme (HEWL) was dissolved in 100 m*M* sodium acetate buffer pH 4.5 to a concentration of 200 mg ml^−1^. Lysozyme powders were prepared by mixing 200 µl buffered lysozyme solution with an equal volume of 100 m*M* sodium acetate buffered pH 4.5 precipitant solution containing 1 *M* sodium chloride. The mixed solutions were stirred constantly at room temperature and microcrystalline precipitate formed within 1 h. In addition to the native lysozyme preparation, ligand-bound and unbound samples were prepared by adding 10 m*M* glucose, NAG or NAG_3_ to the buffered lysozyme solution prior to precipitation. In total, data were collected from three samples of native lysozyme, two samples of lysozyme mixed with glucose, two samples of lysozyme mixed with NAG and three samples of lysozyme mixed with NAG_3_. After 24 h, samples were loaded into 1 mm diameter quartz capillaries and centrifuged for 2–­3 min, during which some of the amorphous precipitates enhanced the packing of polycrystalline material. A small volume of air placed between the powder and the mother liquor was added to keep the powder packed together and the capillaries were flame-sealed to prevent evaporation. All powder samples were 0.5–0.8 mm in length.

Powder diffraction profiles were measured from a sample of native lysozyme on the high-resolution powder diffraction beamline X3B1 (now X16C) at the National Synchrotron Light Source, Brookhaven National Laboratory (Upton, New York, USA). Data were collected at room temperature using a wavelength of 0.6995 Å. The Ge(111) analyser crystal was scanned over a 13° 2θ range (0.64–13.64°) counting each 0.002° step for 4 s. During data collection the sample was continuously rocked by ±10° to ensure adequate powder averaging. No signs of radiation damage were visible by comparison with a second scan taken at the end of data collection. For the ligand-bound/unbound comparison, diffraction profiles were collected using a 0.001° step size for two regions of 2θ: 4–5° and 13–14°. Pearson correlation coefficients were calculated between the raw diffraction profiles of the different samples. It should be pointed out that data sets from each sample were acquired in the first instance before the collection of duplicate and triplicates in order to eliminate potential correlations over the time of data acquisition.

## 3. Results and discussion

**Figure 1:**
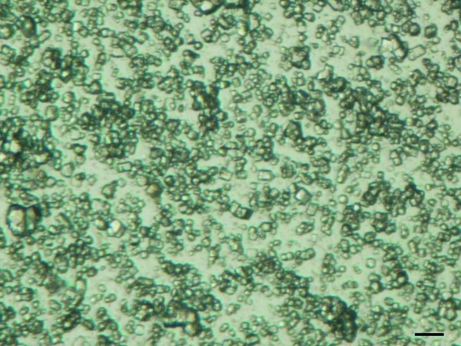
A typical powder sample obtained from precipitation of soluble lysozyme with sodium chloride (scale bar = 50 µm).

A typical lysozyme powder sample is shown in Fig. 1 ▶. The samples are composed of a mixture of single crystals, polycrystalline aggregates and amorphous precipitate. The majority of the single crystals are less than 15 µm in their largest dimension. The shapes of the single crystals are similar to the shapes of larger crystals of the tetragonal form (space group *P*4_3_2_1_2) of HEWL also grown from sodium chloride precipitant in acetate buffer. The small crystals from the powder preparation, like the larger *P*4_3_2_1_2 crystals, are birefringent. Preliminary inspection of all diffraction profiles revealed peaks at 2θ angles consistent with the predicted Bragg reflections calculated using the unit cell (*a* = *b* = 79.3, *c* = 38.0 Å) of the tetragonal form (*P*4_3_2_1_2) of HEWL.

**Figure 2:**
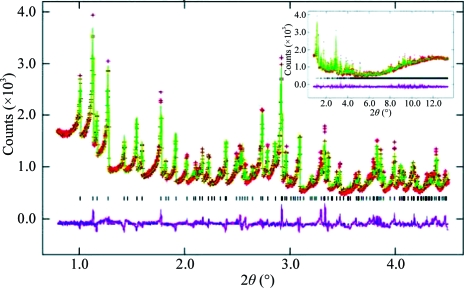
High-resolution powder diffraction profile enlarged for 2θ smaller than 4.5° (the full pattern is shown in the inset) of a sample of soluble lysozyme precipitated with sodium chloride. Observed intensities are shown as red crosses and calculated and differences curves are shown as green and magenta lines, respectively. The second and sixth tick marks of the reflection positions (shown in black) correspond to *hkl* 210 and 201, respectively.

The quality of the lysozyme powder produced was evaluated using stereochemically restrained Rietveld refinement. The protocol used for refinement was similar to the refinement protocol previously described (Von Dreele, 2001 ▶) and used the same lysozyme structure obtained from single crystallography (PDB code 1rfp; Motoshima *et al.*, 1997 ▶) as a starting model. The lysozyme structure was refined against the observed diffraction profile and was consistent with space group *P*4_3_2_1_2. Differences between the calculated and observed profiles are minimal, as shown in Fig. 2 ▶. Even at low resolution, where single non-overlapping reflections are observed, high-intensity reflections (*e.g. hkl* 210) as well as low-intensity reflections (*e.g. hkl* 201) are fitted well from the refinement. The final refined lysozyme structure is consistent with the accepted region of the Ramachandran plot (Ramakrishnan & Ramachandran, 1965 ▶). The refinement converged with a final χ^2^ of 3.85 and root-mean-square deviations on bond lengths and bond angles of 0.012 Å and 1.8°, respectively. These results suggest that the data are of comparable crystalline quality to previously reported work (Von Dreele, 2001 ▶).

**Figure 3:**
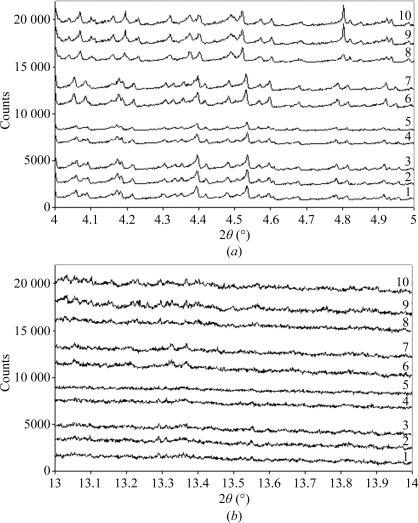
Raw diffraction profiles collected in the 2θ region 4–5° (*a*) and 13–14° (*b*). Samples 1–3 correspond to native lysozyme powder, samples 4 and 5 to lysozyme mixed with glucose, 6 and 7 to lysozyme mixed with NAG and 8–10 to lysozyme mixed with NAG_3_. An arbitrary number of counts were added to the raw intensities for all profiles to generate these overplots, using values of 0, 1500, 3000, 6000, 7500, 9500, 11 500, 14 500, 16 500, 18 500 for samples 1–10 in (*a*) and values of −4500, −3500, −1500, 1500, 4000, 2750, 6250, 9000, 9500, 13 000 for samples 1–10 in (*b*).

**Figure 4:**
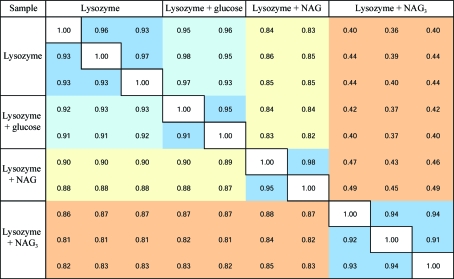
Correlation coefficients were calculated between all raw powder diffraction profiles measured from three samples of native lysozyme, two samples of lysozyme mixed with glucose, two samples of lysozyme mixed with NAG and three samples of lysozyme mixed with NAG_3_. The numbers to the upper right of the diagonal compare pairwise profiles collected in the 2θ range 4–5° corresponding to 10–8 Å Bragg *d*-spacing. The numbers to the bottom left of the diagonal compare pairwise profiles collected in the 2θ range 13–14° corresponding to ∼3 Å Bragg *d*-spacing. The standard Pearson correlation coefficient was used with the 2θ angle assigned as the independent value and the number of counts in the powder diffraction profile assigned as the dependant value.

In the protein ligand-bound/unbound experiment, the 2θ ranges 4–­5° and 13–14° represent regions of the diffraction profile in which minimal and numerous overlapping reflections exist and correspond to Bragg *d*-­spacings of ∼9 and ∼3 Å, respectively. Overplots of all raw diffraction profiles are shown in Fig. 3 ▶. Fig. 4 ▶ presents all the calculated correlation coefficients between raw diffraction profiles when comparing all samples. The correlation between profiles collected in the 4–5° 2θ range is remarkable. The comparison of duplicates and triplicates of samples with correlation coefficients in the range 0.91–0.98 suggest that the diffraction profiles are similar and polycrystalline powder can be grown reproducibly. The correlation coefficients obtained when com­paring native lysozyme and lysozyme + glucose (0.93–0.98) are comparable to those obtained on comparing duplicates/triplicates and suggest that the presence of glucose does not alter the diffraction profile. In contrast, the correlation coefficients obtained in the comparison of lysozyme + NAG with native lysozyme (0.83–0.86) and lysozyme + glucose (0.82–0.84) suggest that the presence of NAG has an effect on the diffraction profile. More striking is the comparison of lysozyme + NAG_3_ with native lysozyme and lysozyme + glucose, which gives correlation coefficients in the range 0.36–0.44 and that are slightly different from those obtained for lysozyme + NAG (0.43–0.49). These results show that the presence of NAG_3_ has an effect on the diffraction profile and that this effect is to some extent comparable to that obtained from lysozyme in the presence of NAG. The correlation coefficients obtained here are in good agreement with the expected binding properties of these compounds to lysozyme. A less obvious but similar pattern is discernable when comparing the correlation coefficients at ∼3 Å (13–­14° 2θ), suggesting that the diffraction profiles measured in this resolution range are also sample-dependant. The higher correlation coefficients on comparing samples at ∼3 Å are most likely to be the result of lower signal to noise and highly overlapping reflections (Basso *et al.*, 2005 ▶; Von Dreele, 2007 ▶).

For further HTS applications, small quantities of sample are required prepared in a high-throughput format. Our initial effort reveals that we can reproducibly grow lysozyme powder in a 96-well plate by mixing 10 µl buffered lysozyme with 10 µl precipitate solution. The diffraction profile acquired by irradiating the lysozyme powder directly through the plate possesses similar characteristics to that collected from samples loaded in capillaries. Furthermore, the data-collection time can be reduced for HTS applications by the utilization of area/strip detectors and a more intense beam. Taken together, these results support the idea that the diffraction profile measured from protein powder samples could be exploited in the development of a generic assay for use in HTS applications and chemical genetics. This approach could exploit the known crystallization conditions of a target for powder preparation, *e.g.* an ‘orphan’ structure solved from a structural genomics effort. It should be pointed out that the approach of comparing raw diffraction profiles would also detect false positives from a change in the unit-cell lattice parameters even if the chemicals do not bind specifically to the protein. Further characterization of the hits using restrained Rietveld refinement could then be achieved. In view of the size of the individual lysozyme crystals used in this study, single crystallography and microdiffraction (Sanishvili *et al.*, 2008 ▶) could also be considered.
